# Single item measures of self-rated mental health: a scoping review

**DOI:** 10.1186/1472-6963-14-398

**Published:** 2014-09-17

**Authors:** Farah Ahmad, Anuroop K Jhajj, Donna E Stewart, Madeline Burghardt, Arlene S Bierman

**Affiliations:** School of Health Policy and Management, Faculty of Health, York University, 4700 Keele Street, HNES 414, Ontario, M3J 1P3 Canada; OPTIMUS | SBR, 30 Adelaide St. E, Suite 600, Toronto, ON M5C 3G8 Canada; Women’s Health Program at University Health Network, University of Toronto, 200 Elizabeth St, Toronto, M5G 2C4 Canada; School of Heath Policy and Management, Critical Disability Studies, Faculty of Health, York University, 4700 Keele Street, Ontario, M3J 1P3 Canada; Lawrence S. Bloomberg Faculty of Nursing; Institute of Health Policy, Management and Evaluation; Dalla Lana School of Public Health and Department of Medicine, Keenan Research Centre in the Li Ka Shing Knowledge Institute, St. Michael’s Hospital, University of Toronto, 30 Bond Street, Toronto, ON M5B 1 W8 Canada

**Keywords:** Self rated mental health, Single item, Review, Measurement

## Abstract

**Background:**

A single-item measure of self-rated mental health (SRMH) is being used increasingly in health research and population health surveys. The item asks respondents to rate their mental health on a five-point scale from excellent to poor. This scoping study presents the first known review of the SRMH literature.

**Methods:**

Electronic databases of Medline, CINAHL, PsycINFO, EMBASE and Cochrane Reviews were searched using keywords. The databases were also searched using the titles of surveys known to include the SRMH single item. The search was supplemented by manually searching the bibliographic sections of the included studies. Two independent reviewers coded articles for inclusion or exclusion based on whether articles included SRMH. Each study was coded by theme and data were extracted about study design, sample, variables, and results.

**Results:**

Fifty-seven studies included SRMH. SRMH correlated moderately with the following mental health scales: Kessler Psychological Distress Scale, Patient Health Questionnaire, mental health subscales of the Short-Form Health Status Survey, Behaviour and Symptom Identification Scale, and World Mental Health Clinical Diagnostic Interview Schedule. However, responses to this item may differ across racial and ethnic groups. Poor SRMH was associated with poor self-rated health, physical health problems, increased health service utilization and less likelihood of being satisfied with mental health services. Poor or fair SRMH was also associated with social determinants of health, such as low socioeconomic position, weak social connections and neighbourhood stressors. Synthesis of this literature provides important information about the relationships SRMH has with other variables.

**Conclusions:**

SRMH is associated with multi-item measures of mental health, self-rated health, health problems, service utilization, and service satisfaction. Given these relationships and its use in epidemiologic surveys, SRMH should continue to be assessed as a population health measure. More studies need to examine relationships between SRMH and clinical mental illnesses. Longitudinal analyses should look at whether SRMH is predictive of future mental health problems.

**Electronic supplementary material:**

The online version of this article (doi:10.1186/1472-6963-14-398) contains supplementary material, which is available to authorized users.

## Background

Short scales to measure physical and mental health of populations are increasingly used in epidemiologic surveys [1] to reduce respondent burden and simplify administration and translation while providing efficient global health indicators. The use of single-item measures is also on the rise. This includes the measure of self-rated mental health (SRMH): “In general, would you say your mental health is: Excellent, Very Good, Good, Fair or Poor?”

The early use of the single SRMH item includes studies conducted in 1970s with college students in relation to personality traits and help-seeking for mental health [2, 3]. In 1981 it was used as part of the National Institute of Health Diagnostic Interview Schedule, which was developed using criteria from the Diagnostic and Statistical Manual of Mental Disorders-III [4]. A single SRMH item was later included in the World Mental Health Composite International Diagnostic Interview [5]. More recently, the SRMH item has been used as a stand-alone indicator of mental health in small and large scale studies. Examples of the national epidemiologic surveys with SRMH as a stand-alone item are the Canadian Community Health Survey and Medical Expenditure Panel Survey [6, 7]. Researchers report significant relationships between SRMH and mental disorders [8], need for care [9], utilization patterns [10] and adherence to treatment plans [11]. Others have examined its relationship with validated clinical measures for diagnosis of mental health conditions [8, 12]. In some instances the SRMH item is used as a construct to validate another mental health measure [13, 14].

A similar single item of self-rated health (SRH) has been used world-wide since the 1950s [15]. SRH asks individuals to rate their health on a 5-point scale ranging from excellent to poor, as in the SRMH. SRH is a strong predictor of mortality [16], health care utilization [17, 18], and morbidity [19]. The parallel wording structure of both items implies that SRMH could potentially measure aspects of mental health as robustly as the physical health indications provided by SRH. Though SRMH is increasingly being used as an indicator of population mental health and as a measure to assess risk for adverse mental health outcomes, less is known about the performance of this item.

Understanding SRMH is important to be able to evaluate previous studies using the item, for application of SRMH as a health indicator, and to provide a basis for future work. Since to our knowledge there are no reviews of SRMH, we initiated a traditional systematic review but the heterogeneity of the published studies using SRMH item didn’t allow us to proceed. Thus, we conducted a scoping study of published studies that have either used or analyzed SRMH. The methodology was informed by the scoping review framework developed by Arksey and O’Malley in 2005 [20]. A scoping review or study is conducted to explore and summarize empirical knowledge in a diverse or heterogeneous area of research. Its primary objective is to produce a descriptive overview of research findings. Thus, the goal of this review was to gain a better understanding of how single item SRMH is used in research and how it correlates with other measures and health outcomes in order to inform the use of this measure in population and public health, health systems improvement, and research.

## Methods

### Search strategy

We searched Medline, CINAHL, PsycINFO, EMBASE and Cochrane Reviews from their inception to July 2012. In consultation with a librarian, a broad search strategy was established using keywords as ‘free terms’. Keyword searches were conducted on titles and abstracts for any of the following word combinations: *self rated mental*, *self perceived mental*, *self assessed mental*, *self reported mental* or *global mental*. The Medline search used following string: ((self adj (reported or assessed or rated or perceived) adj mental) or global mental).tw. An additional strategy searched the aforementioned databases using the keywords *mental health* along with titles of surveys known to use the item: Canadian Community Health Survey (CCHS), Epidemiologic Catchment Area study (ECA), Medical Expenditure Panel Survey (MEPS), National Latino and Asian American Study (NLAAS), National Health Services Postal Questionnaire, Mexican American Prevalence and Services Study (MAPSS), Ontario Health Survey (OHS) Mental Health Supplement, and the World Mental Health Composite International Diagnostic Interview (WMH-CIDI). Manual searches were conducted using the reference sections of identified articles. The search was restricted to English language articles. We did not search grey literature.

### Eligibility criteria

Articles qualified for inclusion if they assessed self-rated mental health, self-reported mental health, self assessed mental health, self perceived mental health, or individual global mental health. The study needed to contain a single item asking for a general mental health rating on a four or five point scale, and results related to this item. Acceptable variations of this question asked about overall mental health, mental health at the present time, mental health, and emotional health (Table 1). Articles were excluded if SRMH was measured using multiple items, or if the single item asked about a specific disorder or a mental health symptom (e.g. depression or worry or downhearted-and-blue) [21].

**Table 1:** **Variations in self-rated mental health item wording**

Item wording	Source(s)
In general, would you say your mental health is:	MEPS, CCHS
1. Excellent	
2. Very Good	
3. Good	
4. Fair	
5. Poor	
How would you rate your overall mental health?	WMH-CIDI, OHS Mental Health Supplement
1. Excellent	
2. Very Good	
3. Good	
4. Fair	
5. Poor	
At the present time, would you say your emotional health is:	ECA
1. Excellent	
2. Very Good	
3. Good	
4. Fair	
5. Poor	
How do you rate your mental health at the present time?	Primary data collection (Peterson et al., 2007)
1. Excellent	
2. Good	
3. Fair	
5. Poor	
How do you rate your mental health?	Primary data collection (Yu et al., 1993; 1997)
1. Poor	
2. Fair	
3. Good	
4. Very good	
5. Excellent	

CCHC: Canadian Community Health Survey.

ECA: Epidemiologic Catchment Area.

MEPS: Medical Expenditure Panel Survey.

OHS: Ontario Health Survey.

WMH-CIDI: World Mental Health Composite International Diagnostic Interview.

We included articles with primary data collection and secondary data analyses. Qualitative studies, literature reviews, and meta-analyses were included but no additional relevant studies were found. The search encompassed all international English articles, and no exclusions were made by sex, gender, ethnicity, geographic location, or age.

### Selection process

Two independent reviewers (FA and AJ) considered abstracts for inclusion or exclusion. A coding scheme was established based on study objectives and the first 15 abstracts in the database. The scheme was pilot tested on the first 30 articles and reviewers achieved 100% consensus. Remaining abstracts were evenly divided for coding. Forty randomly selected abstracts were coded by both reviewers to calculate inter-rater reliability (κ= 0.80). All articles that met the inclusion criteria were considered relevant and retrieved for further review. In the review of full-articles, studies were classified by their objective for using SRMH in a validation study or other studies, which were grouped according to the use of SRMH as a major variable or a minor variable. SRMH was considered a major variable if it was an outcome variable or one of the principal independent variables. Conversely, SRMH was considered a minor variable if it was included as a covariate in models without being discussed in much detail, or if it was not a primary focus in descriptive studies. Studies classified in the minor group [14, 17, 22–35] were tabulated but excluded from synthesis.

### Data synthesis

We extracted information about each study’s design, country of origin, data source, and sample characteristics (sample size, gender distribution, age range, population type and ethnicity). We identified variables that were studied in relation to SRMH, and isolated the results of these analyses. A thematic analysis was conducted through review and re-review of the included articles by the research team until group consensus was achieved. We identified following SRMH research themes: validation; mental health conditions; physical health; use of health services; and social determinants of health. These categories were not mutually exclusive, and some studies fit under multiple themes (e.g. [9, 36]).

## Results

**Figure 1:**
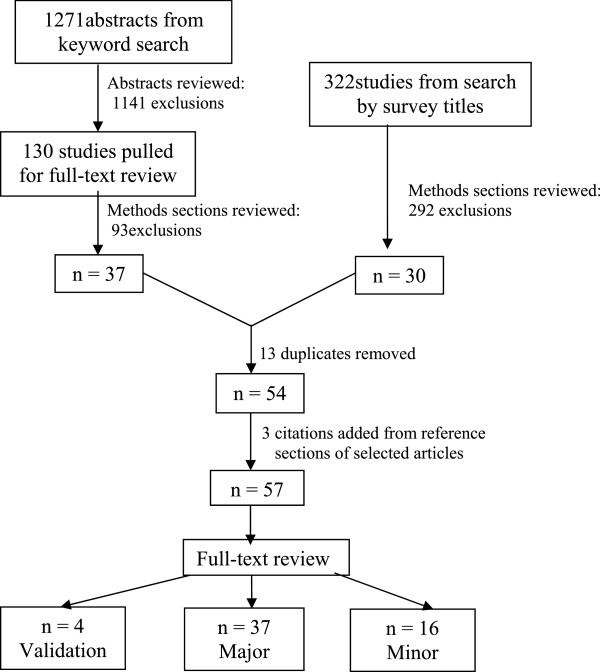
**Study selection flow diagram.**

The literature search found 1271 unique abstracts, 130 of which qualified for full-text review. Thirty-seven articles qualified for inclusion and seventeen new additions were found by searching databases by scales known to contain the SRMH item. Three more articles were found by searching reference sections, for a total of 57 relevant articles. Figure 1 describes the literature review and search process. Please see Additional file 1 for the 57 identified studies.

### Study characteristics

**Figure 2:**
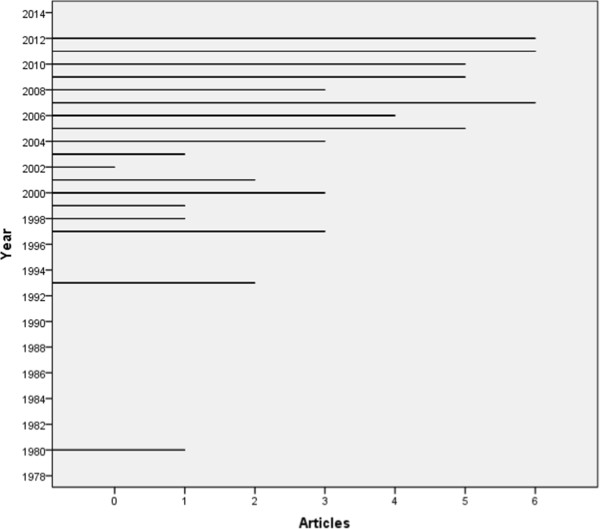
**SRMH articles yearly since 1980.**

Table 2 presents overall study characteristics. While first study on SRMH was published in 1980, additional studies didn’t appear until 1997. SRMH use in research appears to have increased since 2004 (Figure 2). Of the 57 investigations, four examined SRMH in a validation study, 37 used it as a major variable, and 16 included it as a minor variable. Seventeen studies used primary data collection, and the rest were secondary analyses of data from population health surveys. Most of the studies were cross-sectional, while six were prospective. The most commonly used surveys were the Canadian Community Health Survey (CCHS--13 studies), the National Latino and Asian American Study (NLAAS--9 studies) and the Medical Expenditure Panel Survey (MEPS--7 studies). Sixteen studies examined both SRMH and SRH.

**Table 2:** **Self-rated mental health study characteristics**

Characteristic	# of studies	Characteristic	# of studies
	**(N = 57)**		**(N = 57)**
**Country:**		**Sample size:**	
United States	26	<500	8
Canada	20	500–10, 000	32
Other^¥^	11	>10, 000	17
**Data type:**		*Study populations:	
Primary data collection	17	Children ≤12 yrs	0
Secondary data analysis	40	Adults 60+	9
**Study design:**		Individuals with medical conditions^§^	4
Cross sectional	51	Psychological or psychiatric disorders	19
Prospective	6	Mental health service users	5
Case–control	1	University students	2
		Caregivers for the elderly	1
		Veterans	1
*Sex:		General sample	23
Mixed-sex sample	56	**Objective for using SRMH:**	
Male-only sample	1	Validation study	4
Female-only sample	0	Major variable in a research study	37
Analysis of gender differences in SRMH	16	Minor variable in a research study	16
**Secondary data sources used in multiple studies:**		**SRMH as major variable & review themes:**	
Canadian Community Health Survey	13	Validation/ Mental health condition	9
Medical Expenditure Panel Survey	7	Physical health	4
Mental Health Supplement to the Ontario Health Survey	2	Health service (utilization, satisfaction)	13
National Latino Asian-American Study	9	Social determinants of health	19

*Categories under some headings are not mutually exclusive; column totals do not always add to 57.

^¥^Other: China 2, Singapore 2, England 1, Japan 1, Nigeria 1, Puerto Rico 1, Sri Lanka 1, Turkey 1, Ukraine 1.

^§^Medical conditions: Asthma, medically unexplained physical symptoms, multiple sclerosis, restless leg.

Syndrome, hypertension.

Twenty-six studies were conducted in the United States, twenty in Canada, two in China, two in Singapore, and seven in other countries (Ukraine, Japan, England, Nigeria, Puerto Rico, Sri Lanka, and Turkey). Twenty-one studies did not report the ethnic composition of their sample, and the remaining looked at Asian Americans (Chinese, Filipino, Vietnamese, and ‘other’ Asian), Latino Americans (Mexican, Puerto-Rican, Cuban, and ‘other’ Latino), blacks, whites, ‘non-whites’, Turks, Sri Lankans and Nigerians. Twelve of these studies were specific to ethnic minorities, and nine made ethnic comparisons. Nine studies examined adults aged 60+ and none examined children under the age of twelve. Nineteen studies were limited to individuals with psychological or psychiatric disorders, four examined individuals with other medical problems.

Sample sizes ranged from 121 to 120,559. Seventeen studies had over 10, 000 participants. All investigations included men and women, except for one that looked only at men [37]. Thirteen studies did not report the proportion of males and females in their sample, thirteen conducted a gender-based analysis, and sixteen examined gender differences in SRMH.

### SRMH validation studies

Four papers analyzed relationships between SRMH and clinical measures of mental health status. In 1997, Hoff et al. examined prospective data from the Epidemiologic Catchment Area study and found that high scores of SRMH were associated with decreased risk of major depression in the following year. Individuals with poor SRMH were 4.57 times more likely to have a major depressive episode than those with fair SRMH, and 9.97 times more likely than those with excellent SRMH. The effect held constant when controlling for age, gender, or a past history of depression. The authors concluded SRMH could be used to identify groups at higher risk for major depression, even in the absence of other risk factors [8]. Another study reported moderate correlation of SRMH with BASIS-24 [14]. In this study SRMH was a minor variable (see Additional file 1).

In 2007, Fleishman and Zuvekas analyzed MEPS data, a nationally representative US sample. They examined correlations between SRMH, SRH, physical and mental health subscales of the SF-12 health status survey, K6 scale of psychological distress, and Patient Health Questionnaire (PHQ-2) depression screener. The multi-item measures were more strongly correlated with each other (*r* > .69) than with SRMH (correlations ranged between 0.33 and 0.49). SRH correlated more strongly with physical health subscales of the SF-12, and SRMH with mental health subscales of the SF-12, PHQ-2, and K6 [7]. Further, it is important to note that Fleishman and Zuvekas quantified the relationship between SRH and SRMH. They found both variables had a stronger association with each other (*r =* .54) than with other physical and mental health scales. However, SRMH had unique associations with the mental health scales, even when adjusting for SRH. There may be some overlap between what these items are measuring, or a correlation exists between physical and mental health constructs [7].

In 2010, Mawani and Gilmour examined data collected by the CCHS 1.2, Mental Health and Well-Being. The results show consistent and strong association between poor/fair SRMH and several measures of mental morbidity which included WMH-CIDI, self-reported diagnosis of mental disorders, and level of stress. There was a gradient in mean scores of SRMH and prevalence of fair/poor mental health by recency of WMH-CIDI based mental disorders [38]. Jang et al. in 2012 examined a community sample of 420 Korean American older adults residing in the New York City. They found that SRMH was strongly associated with other mental health measures: CES-D (r = .42), GDS-SF (r = .42) and PHQ-9 (r = .50) while controlling for socio-demographics and physical health related variables. These three measures of depressive symptoms made significant contribution to SRMH. The variance explained by the short-form CES-D was 11%, by the GDS-SF was 10%, and by the PHQ-9 was 16% [39].

### SRMH and mental health conditions

Five studies examined SRMH as a major variable in relationship to mental health conditions. In 2006, Olfson conducted a prospective analysis of MEPS data and found that patients with fair or poor SRMH were almost two times more likely to be among the 26.7% of the sample who continued antidepressant therapy beyond 90 days. Results were adjusted for race, age, sex, and pre-treatment mental health status [11]. Study by Tiwari and Wang in 2006 found that Chinese participants were more likely than other Asians or whites to report fair or poor SRMH. However, Chinese and other Asians had lower prevalence rates of mental and substance use-related disorders [6]. In 2008, Zuvekas and Fleishman used MEPS data and compared Whites, Hispanics and Blacks. They found a weaker association between SRMH and emotional symptoms and SRMH and service use for Hispanics and Blacks compared to Whites. They suggested that ethnic differences exist in interpreting emotional symptoms and need for services [9]. In 2011, Kim et al. examined SRMH and psychiatric disorders among non-Hispanic Whites, Blacks, Hispanics, and Asians. They found that non-Hispanic Whites with poor SRMH were more likely to have mood and anxiety disorders [40]. Another study by Kim et al. in 2012 examined the associations between SRMH and diagnoses of psychiatric disorders among American subgroups of Chinese, Filipinos, and Vietnamese origin. The results show that, after controlling for covariates, SRMH was significantly associated with diagnoses for any 12-month DSM–IV psychiatric disorders only among Filipinos (*AOR*: 2.06; 95% CI: 1.29 –3.32) [41]. Ethnicity seems to moderate the relationship between SRMH and mental health conditions.

### SRMH & physical health

Out of four studies using SRMH as a major variable, three examined relationships between poor physical health and fair or poor SRMH and one focused on the quality of life. In 2006, Dogra and Baker examined a group of asthmatics and found that physically active asthmatics had significantly greater SRMH and fewer chronic physical/mental chronic conditions [42]. In 2007, a descriptive study by Park and Knudson report that people with medically unexplained physical symptoms (MUPS) were more likely than those without MUPS to rate their mental health as fair or poor [43]. In 2011, El-Gabalawy examined data from the CCHS 1.2 and found that the comorbidity of anxiety with lung diseases resulted in poor SRMH after adjusting for confounding variables [44].

In 2010, Sawatzky et al. examined SRMH and self-rated physical health as key independent variables in predicting the quality of life (QOL) among Canadian adolescents. They included five life domains (satisfaction with family, friends, living environment, school and self) as mediating variables. The authors found that SRMH, and to a lesser degree self-reported physical health, was significantly associated with differences in satisfaction with five life domains and global QOL. The study also revealed that adolescents differentiated SRMH and self-rated physical health as distinct domains. SRMH was more strongly associated with depressive symptoms, measured by CES-D, than self-rated physical health. The latter was more strongly associated with physical activity than SRMH [12].

### SRMH and use of health services

Thirteen studies examined SRMH as a major variable in relation to health services for its utilization, help-seeking and satisfaction. Of these thirteen, ten studies emphasized help seeking and service use, while three emphasized client satisfaction.

Studies on the utilization of health services examined the use of mental health services, complementary services and general practice in relation to SRMH. In 1997, Katz examined SRMH and the use of outpatient mental health care in Ontario and the US. The odds of receiving any medical or psychiatric or social services for persons with fair or poor SRMH were 2.7 in the US versus 5.0 in Ontario; the difference was eliminated on controlling the perceived need for care. Other control variables included sex, urban location, and age. SRMH was the second highest predictor of service use, preceded by presence of an affective disorder. Anxiety disorders, substance dependence, and comorbid mental health conditions were less predictive [45]. In 2001, Albizu-Garcia et al. examined mental care utilization prospectively among Puerto Ricans and found that poor SRMH and service need were stronger predictors of service use for men than for women [46]. Zuvekas and Flieshman (outlined previously), found fair or poor SRMH was predictive of ambulatory mental health visits and purchasing medications for mental health treatment. Analyses were controlled for sociodemographic factors, health insurance coverage, chronic physical health conditions, supply of psychiatrists with separate regressions including specific mental health scales [9]. The use of complementary services (e.g., chiropractic, acupuncture, massage) was examined by Druss and Rosenheck in 2000. They found the likelihood of using any complementary service did not change with poor or fair SRMH while controlling for age, sex, race, education, total medical/mental conditions, and region. Yet, presence of a mental condition was predictive of the use of complementary service in a similar multivariate model [36]. In 2007, Nabalamba and Miller reported that Canadians reporting fair or poor SRMH were more likely to visit a general practitioner or specialist. A significant association remained after adjusting for age, sex, ability to converse in English or French, household income, urban/rural residence, and having a family doctor [10]. In 2009, Vasiliadis examined CCHS 1.2 dataset for the determinants (grouped as need, enabling and predisposing factors) of visits to family physicians, psychiatrists, psychologists, psychotherapists, and other health professionals for mental health reasons. Among fifteen ‘need based’ significant predictors of service use, SRMH was fifth from the bottom with odds ratio of 1.37 [47]. In multivariable analyses of NLAAS data, Kim et al. in 2010 found that poor self-rated mental health was associated with significantly greater mental health service use among immigrants age 60 and older [48].

Similar findings have been reported by two studies conducted in Singapore. In multivariate analyses examining depressive and anxiety disorders, fair or poor SRMH and acknowledging having a mental illness were predictive of service use for mental and emotional health among adult Singaporeans; health beliefs and social support were not [49]. Another multivariate analysis of Singaporean elderly found fair or poor SRMH was an independent predictor of using mental health services [50].

The relationship between SRMH and help-seeking has been also reported. A descriptive analysis of mental health among Nigerian university students found SRMH was related with SRH, neuroticism, and having problems to discuss with a doctor [2].

### SRMH and experiences with care

Three studies examined SRMH and health service satisfaction. In 2000, Rohland et al. examined American Medicaid patients to determine the relationship between mental health service satisfaction, SRMH, and life satisfaction. The authors found correlations between all three variables for people with schizophrenia but not for those with affective, anxiety or adjustment disorders [51]. Follow-up study is needed to clarify the relationship between service satisfaction and SRMH amongst groups of differing diagnoses to identify whether other variables could be responsible for this effect. In 2007, Raleigh et al. in England found that people with fair or poor SRMH were less likely to be satisfied with mental health services. SRMH was the strongest predictor among all study variables (ethnicity, age, living alone, employment status, and hospital admissions) [52]. Eselius in 2008 found that evaluations of managed behavioural health plans varied by SRMH. Those with excellent or good SRMH gave higher ratings to the plan than those with fair or poor SRMH [53].

### SRMH and social determinants of health

Nineteen studies examined SRMH as a major variable in relation to social determinants of health, such as socio-economic status (e.g., education, income and type of employment), social environments (e.g., family support, community belonging, neighbourhood and nativity), age, gender or ethnicity/race.

Since 1990’s studies have examined SRMH in relation to socioeconomic status along with age, gender and other demographics. In 1993, Yu and Wang examined social status and SRMH in a geriatric outpatient sample in China and found that people with very high and very low levels of education had lower SRMH than blue collar workers, civil servants, and white-collar workers. A potential explanation for low SRMH among highly educated individuals hypothesized to be the effect of the communist regime [54]. In 1997, Yu et al. analyzed this dataset to examine social factors in relation to SRH and SRMH. The predictors of low SRMH were old age, perceived lack of family respect, number of diseases, conflictual neighbourhood relations, percentage of income spent on rent, unmet preference to live with a son, and personal monthly income [55]. In 2000, Druss and Rosenheck found respondents with fair or poor SRMH were older, less likely to have a high school education, and had more self-reported mental or physical conditions [36]. In 2000, O’Donell examined SRMH among veterans and non-veterans and found lower SRMH for the latter but this group difference was eliminated on controlling for demographic, socioeconomic, and health-related factors [37]. In 2005, Cohen and Patten found a gender effect among Alberta medical residents; more males reported excellent SRMH than female residents. Overall 17% of residents reported fair or poor SRMH compared to 8% in national community health survey [56]. In 2006, Shields found that low satisfaction with job was related to fair or poor SRMH [57]. In 2008, Zuvekas and Flieshman found poorer SRMH among those who had a lower income, were less educated, female, or aged 41–60 [9]. In 2010, De Castro examined NLAAS data finding that employment frustration was associated with low SRMH even after controlling for gender, age, ethnicity, education, occupation, income, whether immigrated for employment, years in the United States, English proficiency, and a general measure for everyday discrimination [58]. In 2010, Maximova et al. examined resettlement experiences of Canadian refugees and found that having employment and access to settlement services were associated with improvements in SRMH while time spent in a refugee camp and having held a professional job in home country were associated with a decline in SRMH [59].

Some studies have examined the relationship between SRMH and the social environment. In 2005, Statistics Canada released a report on community belonging and self-perceived health using CCHS dataset. Stronger feelings of belonging were associated with substantially better SRMH and self-rated physical health [60]. In 2007, Mulvaney-Day et al. analyzed NLAAS dataset to examine the relationships between social support, social cohesion, and self-rated physical health and SRMH in a sample of Latinos. They found that family support was strongly associated with positive SRMH after controlling for language, education, income and other demographics [61]. In 2009, Zhang analyzed NLAAS data to examine the role of social connections (i.e. family cohesion, relative support, friend support, and neighborhood cohesion), socioeconomic status, and immigration-related factors on the self-rated physical and SRMH. The four types of social connections were all related to SRMH but family cohesion had independent and direct effects on SRMH over and above controls and mediators [62]. A multivariate analysis looking at neighbourhood environment and SRMH in Southern Sri Lanka found that environmental stressors (nuisance from neighbours or drug users, shortage of water, or having poor water/ sewage drainage system) were associated with fair or poor SRMH, but not with fair or poor SRH [63]. In 2012, three studies were published using NLASS data, assessing the relationship between SRMH and nativity. Within a sample of first-generation Asian Americans, Lam et al. examined SRMH and the effects of age of immigration, age, and perceived difference on social status. There was no significant effect on SRMH due to age and age of immigration. However, when perceived difference in social status was considered, then age had a bearing on both physical and mental health [64]. Schachter et al. found that use of both English and ethnic language was associated with better self-rated physical health and SRMH; associations were partially mediated by socioeconomic status and family support [65]. In 2012, John et al. examined the associations of nativity and occupational class with SRMH while controlling for age and gender. Comparing U.S. born-Asians and immigrant Asians, John et al. found that immigrants were more likely to report fair or poor SRMH (adjusted OR 2.6) though less likely to report mental disorder and anxiety (adjusted odd ratio 0.6). No gradient was found between occupational class and SRMH within Asian immigrants, unlike the U.S. born Asians [66].

Several studies examined differences in SRMH associated with race and ethnicity. Using MEPS data, Zuvekas and Fleishman found Blacks and Hispanics were more likely than Whites to report excellent SRMH, and less likely to report poor SRMH (even when they had low scores on the mental component summary of the SF-12). In 2011, Veenstra examined data from Toronto and Vancouver. Associations were examined between SRMH, SRH and self-identified racial identities (i.e., Asian, Black, South Asian, and White). Respondents expressing Asian identity reported poorer SRMH and SRH, which were not explained by their socio-economic status [67]. Additional studies examining ethnic and racial differences are described under theme of mental health conditions.

SRMH has also been examined in relationship to care giving or smoking. A prospective study of caregivers for the elderly found SRMH to have declined during the 2-year study period. Decline in SRMH was predicted by poor baseline SRMH and decline in SRH [68]. A study of smoking among Nova Scotians used SRMH as a minor variable and found that more people with poor SRMH smoked compared to those with poor SRH [35].

## Discussion

Our scoping review of the literature found 57 studies providing information on the performance of SRMH in diverse contexts. SRMH correlated moderately with mental health scales, but there are ethnic differences in responses to the item. Poor SRMH was associated with poor SRH, physical health problems, increased health service utilization, and a lower likelihood of being satisfied with mental health services. Some studies found age and gender disparities in SRMH but others did not. While few studies conducted formal validations of SRMH, its use in the literature gives us important information about its relationship with other variables.

This study is the first review of SRMH literature. Given the increasing use of this item in the recent years, it is important to understand the scientific contributions made by this item and the strengths and weaknesses in its use. We optimized the number of articles we found by using a broad, structured search strategy. However, a limitation of our research is that SRMH terminology is not yet standardized, and some studies may not have been captured by the selected keywords. We tried to minimize this limitation by conducting a second search of surveys known to include SRMH. A limitation of the SRMH research literature is that heterogeneity precludes meta-analyses. The review focused on single item SRMH, analogous to the single item SRH. Thus, studies that measure other aspects of mental health by using other single items were excluded.

The moderate correlations between SRMH and mental health scales indicate these measures are related but not interchangeable. SRMH may be measuring factors outside the scope of mental health scales, but based on current literature it is unclear what these other factors are. Given the similar wording and correlation between SRMH and SRH, we may be able to look toward SRH literature for hypotheses. When SRH was first studied, researchers found moderate correlations between SRH and current health status. When longitudinal studies were conducted, SRH was shown to be an equal or stronger predictor of mortality, morbidity, and utilization than many commonly used measures. Similarly, SRMH may be capturing developing mental health problems, in addition to existing disorders. Studies included in this scoping review have shown relationships between SRMH and health service utilization. Hoff et al. (1997) have demonstrated that SRMH can predict the risk of future depression. More investigation is needed to fully understand these relationships. While single items have the advantage of simplicity and ease of administration, they cannot capture complexities assessed using multi-item scales, and like other instruments may result in false positives and negatives. However, they can provide important information. The use of SRMH in epidemiologic and health surveys and the relationships identified in this review indicate that SRMH could become a robust population mental health measure. It has potential to provide estimates of the mental health of populations, be used to assess change over time in response to changes in policy and practice. SRMH may also hold value as a screener to identify individuals and populations at risk for future mental health problems, but research is limited thus far.

## Conclusions

SRMH is seeing increased use in research and in population health surveys. This scoping review points to a number of relationships between SRMH and mental, physical, social, and utilization variables. SRMH may also be predictive of mental morbidity. However, more work needs to be done before these relationships can be firmly established. Future research should continue to further define the relationship between SRMH and measures of mental health or specific disorders. More longitudinal research is needed to determine whether SRMH is predictive of future mental health conditions. Studies should also look at how SRMH varies by sociodemographic characteristics (sex, ethnicity, age, socioeconomic status). In addition, more information is needed about how different population subgroups respond to this item, particularly if it is to be used to assess disparities. Finally, qualitative analysis could be useful in understanding individual response mechanisms behind this item.

## Electronic supplementary material

Additional file 1: Table 3: Studies Using Self-Rated Mental Health (arranged thematically). (DOC 151 KB)
