# Supplementary material for: Single item measures of self-rated mental health: a scoping review
Source: BMC Health Serv Res. 2014 Sep 17;14:398. doi: 10.1186/1472-6963-14-398 (PMC4177165; doi:10.1186/1472-6963-14-398)
Supplement: Supplementary file 1 — Additional file 1: Table 3: Studies Using Self-Rated Mental Health (arranged thematically). (DOC 151 KB) [file 12913_2014_3498_MOESM1_ESM.doc]

| **TABLE 2.3.** Studies Using Self-Rated Mental Health (arranged thematically) |
| --- |

| **Yr** | | **Study** | | **Theme** | **Objective*** | | | **Data Source** | | | **Sample** | **MH Variables studied with SRMH** | | **Other Variables studied with SRMH** | | | | **Findings in relation to SRMH** |  | | |
| --- | --- | --- | --- | --- | --- | --- | --- | --- | --- | --- | --- | --- | --- | --- | --- | --- | --- | --- | --- | --- | --- |
| 1997 | | Hoff, R.A., *et al.*  Prospective | | Validation | | 3 | Epidemiologic Catchment Area Study (1980) | | | USA  n = 3,684  age: 18+  m/f: 3/4  dx: MH  general pop’n | | | Measures of subclinical depression, previous or current depression diagnosis | Demographics, social status, physical health, social support, health care utilization | | Positive SRMH was associated with decreased risk of a depressive episode in following year (adjusted for age, gender& depression history). | | |  | | |
| 2007 | | Fleishman, J.A., & Zuvekas, S.H.  Cohort | | Validation | | 3 | MEPS (2004) | | | USA  n = 11,109  age: 18+  m/f: 1/2  dx: other  general pop’n | | | SF-12 Health Status Survey, K6 scale of non-specific psychological distress, PHQ-2 depression screener | SRH, socio-demographic characteristics, medical conditions | | K6, PHQ-2 & MH subscales of the SF-12 were more strongly correlated with each other (r > .69), than with SRMH (r ≈ .33 to .49). Authors concluded SRMH reflects psychologic distress or depression, but not exclusively. | | |  | | |
| 2010 | | Mawani, F., & Gilmour, H.  Cross-sectional | | Validation | | 3 | CCHS 1.2 (2002) | | | Canada  n = 36, 984  age: 15+  m/f total: not given  dx: MH  dx: other  general pop’n | | | Agoraphobia, bipolar disorder, depression, panic disorder, social phobia (WHM-CIDI) | Socio-demographic factors; immigrant status, chronic physical conditions | | In 2002, 7% of Canadians rated their mental health as fair or poor. Respondents classified with mental morbidity reported lower SRMH. | | |  | | |
| 2012 | | Jang, Y., *et al.*  Cross-sectional | | Validation | | 3 | Primary data collection | | | US  n = 433  age: 60-98  m/f: approx. 1/1  Korean-Amer pop’n | | Depressive symptoms: CES-D, GDS-SF, PHQ-9 | | Socio-demographic characteristics, physical health | | | Three measures of depressive symptoms were interrelated; each made a significant contribution to SRMH. | | |  | |
| *Objective for using SRMH: “1” as a minor variable; “2” as a major variable; “3” as the subject of a validation study | | | | | | | | | | | | | | | | | | | |  | |
| 2004 | | Eisen, S.V., *et al*.  Prospective | | Mental conditions | | 1 | Primary data collection | | | USA  n = 5,878  age: 18+  m/f: 1/1  dx: MH or substance abuse Rx | | BASIS-24© summary & subscale scores, SF-12 | | Inpatient or outpatient status | | | Correlation between BASIS-24© summary score & SRMH was 0.65 for inpatients, 0.75 for outpatients. Highest subscale correlation was for depression/ functioning. | | |  | |
| 2004 | | Shields, M.  Cross-sectional | | Mental conditions | | 1 | CCHS 1.2 (2002) | | | Canada  n = 36,984  age: 15+  m/f total: not given  general pop’n | | Social anxiety disorder | | SRH | | | People with social anxiety disorder had lower ratings of SRMH & SRH | | |  | |
| 2005 | | Harman, M., *et al*  Cross-sectional | | Mental conditions | | 1 | MEPS (2000-2001) | | | USA  n = 498  age: 65+  m/f: not given  dx: MH  dx: other  pop’n: older American | | Depression, adequacy of depression treatment (number of psychotherapy sessions, anti-depressant refills) | | chronic medical conditions (heart disease, diabetes, arthritis, hypertension) | | | SRMH, race, supplemental insurance, income, education, sex & marital status were adjusted for to determine odds of receiving adequate depression care across 4 chronic conditions. | | |  | |
| 2006 | | Olfson, M., *et al.*  Prospective | | Mental conditions | | 2 | MEPS (1996-2001) | | | USA  n = 829  age: 18+  m/f: approx. 1/1  dx: MH  pop’n: dx depression | | Antidepressant use, type of antidepressant | | SRH; socio-demographic and socio-economic factors; service access; ethnicity | | | Continuing anti-depressant treatment beyond 90 days was associated with f/p SRMH; f/p SRH; treatment with an SSRI or SNRI. Results adjusted for age, race, sex & pre-treatment MH. | | |  | |
|  | | *Objective for using SRMH: “1” as a minor variable; “2” as a major variable; “3” as the subject of a validation study | | | | | | | | | | | | | | | | | |  | |
| 2006 | | Tiwari, S.K., Wang, J.L.  Cross-sectional | | Mental conditions | | 2 | CCHS 1.2 (2002) | | | Canada  n = 35,245  age: 15+  mean age: 42.2  m/f: 1/1  general pop’n | | WMH-CIDI diagnosis of a mental or substance use disorder; DSM-IV; CIDI-SF. | | Ethnicity (white, Chinese, other Asian) | | | Chinese were more likely to have f/p SRMH than Whites or other Asians. Chinese & other Asians had lower prevalences of mental & substance use disorders (prevalences were similar to China’s) | | |  | |
| 2008 | | Zuvekas, S.H. & Fleishman, J.A.  Cohort | Mental conditions  Health service utilization  Social determinants | | | 2  2  2 | MEPS (2000-2004) | | | USA  n = 36,459  age: 18+  m/f: 1/2  general pop’n | | SF-12 mental component summary (MCS) for emotional symptoms, MH ambulatory visits, psychotropic medication usage | | Ethnicity, gender, region, socio-demographic, medical conditions | | | SRMH related to ambulatory MH treatment visits & purchase of drugs for MH conditions. A weaker association was found between SRMH and MCS, and SRMH and service use for Hispanics and Blacks compared to Whites. Women, the elderly, low-income & less educated were more likely to report poor SRMH | | |  | |
| 2009 | | Olfson, M., *et al*.  Prospective | | Mental conditions | | 1 | MEPS (1996, 2005) | | | USA  n = 47,438  age: 6+  m/f: 1/1  dx: MH  general pop’n | | National rate of anti-depressant use, MH service use | | Ethnicity, year of treatment, medical history | | | SRMH, age, sex, ethnicity, annual family income & insurance status were adjusted to measure rate of anti-depressant use for 1996 & 2005. | | |  | |
| 2011 | | Morin, C., *et al.*  Cross-sectional | | Mental conditions | | 1 | Primary data collection | | | Canada  n = 2000  age: 18-99  mean age: 48.6  m/f: 1/1  general pop’n | | DSM-IV, Insomnia | | SRPH; socio-demographic and socio-economic factors; gender | | | Insomnia associated with female sex, older age, and poorer SRMH and SRH. | | |  | |
|  | | *Objective for using SRMH: “1” as a minor variable; “2” as a major variable; “3” as the subject of a validation study | | | | | | | | | | | | | | | | | |  | |
| 2011 | | Tintle, N., *et al.*  Cross-sectional | | Mental conditions | | 1 | Primary data collection | | | Ukraine  n =1843  age: 50-91  m/f: approx. 1/1.5  dx: MH  elderly pop’n with depression | | CIDI – 3.0 for depression, mental health history | | Demographic factors, gender, alcohol history, poverty, self-care and cognitive impairment, SRPH | | | Poor SRMH and SRPH and history of major depressive episode (MDE) before age 50 significantly associated with MDE. | | |  | |
| 2011 | | Kim, G., *et al.*  Cross-sectional | | Mental conditions | | 2 | Collaborative Psychiatric Epidemiologic Surveys (2002) | | | USA  n=1,840  age: 60+  m/f: 1/1.4  diverse elderly pop’n | | WMH-CIDI manual for psychiatric disorders | | Ethnicity; socio-demographic factors | | | People who reported poor SRMH and were non-Hispanic Whites were more likely to have mood and anxiety disorders*.* | | |  | |
| 2012 | | Kim, G., *et al.*  Cross-sectional | | Mental conditions | | 2 | NLAAS (2002) | | | USA  n =1639  age:18+  mean age: 43  m/f: 1/1.3  Asian-American pop’n | | WMH-CIDI manual for psychiatric disorders | | Length of residence in US, English proficiency, socio-demographic factors | | | SRMH was significantly associated with diagnoses for any 12-month DSM–IV psychiatric disorders among Filipinos, and not for Chinese and Vietnamese, after controlling for confounders | | |  | |
| 2003 | | Sevim, S. ,*et al*.  Cross-sectional | | Physical health | | 1 | Primary data collection | | | Turkey  n = 3, 234  age: 18-79  mean: 40.48  m/f:1/1  dx: other  general pop’n | |  | | SRPH, restless leg syndrome, age, location, gender, history, comorbid illnesses, lifestyle, socio-economic factors | | | Those with restless leg syndrome had worse SRMH & SRH than individuals without restless leg syndrome. | | |  | |
|  | | *Objective for using SRMH: “1” as a minor variable; “2” as a major variable; “3” as the subject of a validation study | | | | | | | | | | | | | | | | | |  | |
| 2006 | | Dogra, S. & Baker, J.  Cross-sectional | | Physical health | | 2 | CCHS 2.1 (2003) | | | Canada (asthmatics)  n = 11, 243  median age: 40-44  m/f: 1/1.1  dx: other | | Use of mental health services, assessed need | | SRH, physical activity, other chronic conditions | | | Physically active asthmatics had greater SRMH & SRH than those who were inactive. | | |  | |
| 2007 | | Park, J. Knudson, S.  Cross-sectional | | Physical health | | 2 | CCHS 2.1 (2003) | | | Canada  n = 2,562  age: 12+  m/f: not given  general pop’n  dx: MH  dx: other | | Recent major depressive disorder, bipolar disorder, panic and social anxiety disorders, agoraphobia | | SRH, medically unexplained physical symptoms (chronic fatigue, fibromyalgia, multiple chemical sensitivity), gender, socio-demographic and socio-economic factors | | | Those with medically unexplained physical symptoms were more likely to have f/p SRMH & SRH. | | |  | |
| 2007 | | Peterson, E. W., *et al*  Cross-sectional | | Physical health | | 1 | Primary data collection | | | USA  n = 1,064  age: 45-90  m/f:=approx.1/3  dx: other  Older pop’n with multiple sclerosis | |  | | SRH; fear of falling, activity curtailment, years since diagnosis | | | People with multiple sclerosis reporting less than excellent SRMH were more likely to report activity curtailment due to a fear of falling. | | |  | |
| 2010 | | Sawatzky, R. *et al.*  Cross-sectional | | Physical Health | | 2 | Primary data collection | | | Canada  n = 8,225  age: 12-18  m/f: 1/1  adolescent pop’n | | Depressive symptoms: CES-D | | SRPH, global Quality of Life (QOL), Students’ Life Satisfaction Scale for five domains: family, friends, school, environment and self | | | SRMH, and to a lesser degree SRPH, was significantly associated with differences in satisfaction with five life domains and global QOL.  Compared to SRPH, SRMH more strongly associated with CES-D. | | |  | |
|  | | *Objective for using SRMH: “1” as a minor variable; “2” as a major variable; “3” as the subject of a validation study | | | | | | | | | | | | | | | | | |  | |
| 2011 | | El-Gabalawy, R., *et al.*  Cross-sectional. | | Physical health  Mental health | | 2  1 | CCHS Cycle 1.2 (2002) | | | Canada  n = 12, 792  age: 55+  m/f: 1/1  general pop’n | | WMH-CIDI manual for anxiety disorders | | Comorbid physical health conditions, SRPH, socio-demographic factors | | | Comorbidity of anxiety with physical health conditions results in poorer SRMH and SRPH. | | |  | |
| 2012 | | Ohtsu, T., *et al*  Cross-sectional | | Physical health | | 1 | Primary data collection | | | Japan  n =1224  age: 20+  mean age: 51  m/f: 1/1.25  general pop’n | |  | | SRH, relaxation and recreation | | | Insufficient rest and free time had positive independent associations with poor physical and mental health. | | |  | |
| 1980 | | Jegede, R. O.  Cross-sectional | | Health service, help-seeking | | 2 | Primary data collection | | | Nigeria  n = 870  age ~20-35  mean age: 24.75  m/f: 5/1  pop’n: university students | | Neuroticism (i.e. anxiety) | | SRH, having problems to discuss with a doctor; frequency of doctor consultations | | | SRMH was related with neuroticism, SRH & having problems to discuss with a doctor. | | |  | |
| 1993 | | Watkins, A.J. & Kligman, E.W.  Case control | | Health service, utilization | | 1 | Primary data collection | | | USA  n =224  age: 60+  m/f: not given  elderly pop’n | |  | | SRH, attendance to a health-promotion program; health limitations; socio-economic factors | | | Attendance to the seniors’ health-promotion program was not affected by SRMH or SRH. | | |  | |
| 1997 | | Katz, S.J., *et al.*  Cross-sectional | | Health service, utilization | | 2 | Ontario Health Survey, Mental Health Supp. | | | Canada, USA  n = 11,654  age: 18-54  m/f: not given  general pop’n | | Use of medical, psychiatric or social services, mental disorders (affective, anxiety, substance dependence, comorbid conditions) | | Perceived need (self-motivated visit for MH services, feeling need to seek help in past 12 months) | | | Relationship between f/p SRMH & medical/ psychiatric service use was twice as strong in Ontario than USA. Fair/poor SRMH was 2^nd^ best predictor of service use, preceded by having an affective disorder. Controlled for perceived need eliminated differences between countries. Adjusted for age, sex, urban location & country. | | |  | |
|  | | *Objective for using SRMH: “1” as a minor variable; “2” as a major variable; “3” as the subject of a validation study | | | | | | | | | | | | | | | | | |  | |
| 1998 | | Lin, E., *et al.*  Cross-sectional | | Health service, utilization | | 1 | MH Supplement to the Ontario Health Survey | | | Canada  n = 9, 953  age: 15+  m/f: not given  general pop’n | | Having a mental disorder | | Reported disability, gender, age, region, health care spending | | | SRMH, having a mental disorder & reported disability were combined in a single variable representing need. | | |  | |
| 2000 | | Druss, B.G., *et al.*  Cross-sectional | | Health service, utilization  Social det-erminants | | 2  1 | MEPS (1996) | | | USA  n = 16,038  age: 18+  m/f: not given  general pop’n | | MH conditions (psychiatric disorder, affective, anxiety, substance use, adjustment, other) | | Use of compli-mentary medicine (e.g. chiropractic, massage, nutritionist); medical conditions | | | SRMH was not associated with an increase in use of complimentary services. Having a mental condition was predictive of service use when controlling for fair/poor SRMH, chronic medical conditions, total number of conditions, & demographic variables. | | |  | |
| 2000 | | Rohland, B.M., *et al*.  Cross-sectional | | Health service, satisfaction | | 2 | Primary data collection | | | USA  n =815  age: 18-64  mean age: 37+/-11.4  m/f: 1/2.3  dx: MH | | Schizophrenia, affective disorders, anxiety disorders, adjustment disorders | | Service satisfaction, life satisfaction | | | Service satisfaction was correlated with SRMH among persons with schizophrenia but not among those with affective or anxiety disorders. | | |  | |
| 2001 | | Vega, W. A., *et al.*  Cross-sectional | | Health service, utilization | | 1 | Mexican American Prevalence and Services Study | | | USA  n = 507  age: 18+  m/f: not given  Mexican American pop’n  dx: MH | | Having 1 or more DSM III disorder | | Birth in America or Mexico, use of physician or MH provider | | | Poor SRMH among American-born Mexicans had a much greater effect of increasing medical provider use than among Mexican-born immigrants. | | |  | |
| *Objective for using SRMH: “1” as a minor variable; “2” as a major variable; “3” as the subject of a validation study | | | | | | | | | | | | | | | | | | | |  | |
| 2001 | | Albizu-Garcia, C.E., *et al.*  Prospective | | Health service, utilization | | 2 | Mental Health Care Utilization Among Puerto Ricans (1992-94) | | | Purerto Rico  n = 3,221  age: 18-69  m/f: 1/1.5  sample from low socio-economic neighbourhoods | | MH service utilization | | Gender | | | Gender alone was not predictive of MH service utilization. The interaction between gender & SRMH was predictive of service utilization for men. | | |  | |
| 2005 | | Bergeron, E.P. *et al*  Cross-sectional | | Health service, utilization | | 1 | CCHS 1.2 (2002) | | | Canada (youth with mental disorders)  n = 1,092  age: 15-24  m/f: approx 1/1  pop’n: young Canadians  dx: MH | | Mood, substance or anxiety disorder, use of MH services (hospitalization, psychiatrists, psychologists, GPs, nurses, social workers, support  groups, help lines, alternative care,  clergy); WMH-CIDI, K10 | | SRPH, socio-demographic and socio-economic factors, urban/rural, ability to do day-today activities. | | | Youth with f/p SRMH were 4 times more likely to use any MH service during the 1-year period than were those who perceived it as excellent. | | |  | |
| 2005 | | Vasiliadis, H.*, et al.*  Cross-sectional | | Health service, utilization | | 1 | CCHS 1.2 (2002) | | | Canada  n = 36,984  age: 15+  m/f: not given  general pop’n | | Use of MH services (GP, psychiatrist, psychologist, other professional, volunteer support); regional differences | | Provinces and territories | | | SRMH and SRH were combined and entered in the model along with other conditions. Presence of a mental health condition was the consistent predictor | | |  | |
| *Objective for using SRMH: “1” as a minor variable; “2” as a major variable; “3” as the subject of a validation study | | | | | | | | | | | | | | | | | | | |  | |
| 2007 | | Nabalamba, A. & Miller, W.  Cross-sectional | | Health service, utilization | | 2 | CCHS 3.1 (2005) | | | Canada  n = 120,559  age: 18+  m/f: not given  general pop’n | | - | | SRH, number of chronic conditions, visits to a GP or specialist, age, gender, visible minority, socio-economic factors | | | Those with f/p SRMH were more likely to visit a GP, visit a GP 4+ times, or visit a specialist. Association existed for SRH as well. Controlled for age, sex, language, income, urban/ rural residence & having a family doctor. | | |  | |
| 2007 | | Raleigh, V., *et al.*  Cross-sectional | | Health service, satisfaction | | 2 | Primary data collection | | | England  2 surveys:  n =27,398  n = 26,555  age: 16-64  m/f: 1/1.3  pop’n: MH service users  dx: MH | | MH service satisfaction | | Ethnicity, age, living alone, employment status, hospital admissions | | | Those with f/p SRMH were less likely to be satisfied with MH services. SRMH was the strongest predictor of all the study variables. | | |  | |
| 2008 | | Ng, T., *et al.*  Cross-sectional | | Health service, utilization | | 2 | National Mental Health Survey of Adults in Singapore (2003-04) | | | Singapore  n = 2,801  age: 20-59  mean age: 41+/-9.6 yrs  m/f: 1/1.7  general pop’n | | Acknowledgement of having a mental illness | | Health beliefs, social support, service utilization (GP, psychologist, psychiatrist, social worker, MH counsellor) | | | F/p SRMH & acknowledging having a mental illness were predictive of MH service use, health beliefs & social support were not. | | |  | |
| 2008 | | Eselius, L.L., *et al.*  Cross-sectional | | Health service, satisfaction | | 2 | Experience of Care and Health Outcomes Survey (2001) | | | USA  n = 4,068  age: 18+  m/f: 1/4  dx: MH  pop’n: enrolled in behavioural pgm. | |  | | SRH, evaluation of managed behavioural health plan, ethnicity, socio-economic factors | | | Evaluations of managed behavioural health plans were lower for those with f/p SRMH (compared to those with more positive SRMH). | | |  | |
| *Objective for using SRMH: “1” as a minor variable; “2” as a major variable; “3” as the subject of a validation study | | | | | | | | | | | | | | | | | | | |  | |
| 2009 | | Nyunt, M.S.Z., *et al*  Cross-sectional | | Health service, utilization | | 2 | National Mental Health Survey of Elderly in Singapore (2003) | | | Singapore (Singaporean elderly)  n = 1,092  age: 60+  dx: MH  pop’n: eldery w mental disorder | | Disability from mental illness | | MH service utilization (GP, psychologist, psychiatrist, social worker, MH counsellor), gender, socio-economic factors | | | F/p SRMH was an independent predictor of MH service utilization. | | |  | |
| 2009 | | Vasiliadis, H. *et al.*  Cross-sectional | | Health service, utilization | | 2 | CCHS 1.2 (2002) | | | Canada  n = 35, 236  age: 18+  age mean: 45.6  m/f: not given  general pop’n | | Presence of chronic conditions, depression and panic attacks; unmet mental health needs; psychological well-being | | Outpatient service use, SRH | | | SRMH was fifth from bottom among fifteen ‘need based’ significant predictors of service use. | | |  | |
| 2010 | | Kim, G., *et al.*  Cross-sectional | | Health service, utilization | | 2 | NLAAS (2002) | | | USA  n = 501  age: 60+  m/f: 1/1.2  pop’n: elderly immigrant | | Mental health service use, MH need factors | | Ethnicity; predisposing factors (socio-dem); enabling factors (#yrs in US, English proficiency | | | Poor SRMH was associated with significantly greater mental health service use. | | |  | |
| 2011 | | Kim, G., *et al.*  Cross-sectional | | Health service, utilization | | 1 | NLAAS (2002) | | | USA  n = 372  age: 18-86  age mean: 43  m/f: 1/1.6  dx: MH  pop’n: immigrants with mental illness | | Mental health service use, diagnosed psychiatric illness | | English proficiency, health insurance | | | Limited English proficiency decreased odds of mental health service use among Latino immigrants. | | |  | |
| *Objective for using SRMH: “1” as a minor variable; “2” as a major variable; “3” as the subject of a validation study | | | | | | | | | | | | | | | | | | | |  | |
| 1993 | | Yu, L.C. &  Wang, M  Cross-sectional | Social determinants | | | 2 | Primary data collection | | | China  n = 213  age: 65-94  m/f: 1/1.3  pop’n: Chinese elderly | |  | | Job type (blue collar, white collar, civil servant, intellectual), illiteracy, SES, SPPH, disease patterns, gender | | | Chinese intellectuals & illiterates had lower SRMH than blue collar workers, civil servants & white-collar workers | | |  | |
| 1997 | | Yu, L.C.*, et al.*  Cross-sectional | Social  determinants | | | 2 | Primary data collection | | | China  n = 233  age: 65-94  m/f: 1/1.3  pop’n: Chinese elderly | |  | | SRPH, age, perceived family respect, number of diseases, neighbourhood relations, percentage of income spent on rent, preference to live with a son, monthly income, gender. | | | Predictors of low SRMH were age, perceived family respect, number of diseases, neighbourhood relations, percentage of income spent on rent, preference to live with a son & personal monthly income | | |  | |
| 1999 | | Haug, M.R.  Prospective | Social determinants | | | 2 | Medicare Use of Services Survey | | | USA  n = 121  age: <45->75  majority: 65+  m/f: 1/1.6  pop’n: identified caregivers | | Change in SRMH | | SRPH, change in SRPH, providing care for elderly, social isolation, gender, ethnicity, socio-dem factors | | | SRMH & SRH declined over course of study. Predictors of decrease in SRMH: poorer MH to begin with, decline in SRH | | |  | |
| 2000 | | O’Donnell, J.C.  Cohort | Social determinants | | | 2 | MEPS (1996) | | | USA  n = 1,068  age 65+  male only  pop’n: men 65+ | |  | | Veteran or non-veteran, socio-economic factors, health status, mobilization | | | Military service was not a risk factor for poorer SRMH (after controlling for demographic, socioeconomic & health-related factors) | | |  | |
| *Objective for using SRMH: “1” as a minor variable; “2” as a major variable; “3” as the subject of a validation study | | | | | | | | | | | | | | | | | | | |  | |
| 2004 | | Nova Scotia Department of Health  Cross-sectional | Social determinants | | | 1 | CCHS 2.1 (2002) | | | Canada  n = 36,984  age: 12+  m/f: not given  general pop’n | |  | | SRH, Smoker/non-smoker, level of education; socio-economic status. | | | 58% of those with poor SRMH smoke, while 31% of those with poor SRH smoke | | |  | |
| 2005 | | Cohen, J.S. &  Patten, S.  Cross-sectional | Social determinants | | | 2 | Primary data collection | | | Canada  n = 415  age: 24-49  m/f: 1/1  pop’n: Alberta med residents | | Perceived stress; understanding and need for well-being resources | | Gender, being in a medical residency | | | Medical residents had lower SRMH than rest of Canada. More male residents rated their MH as excellent than females (which also occurred in the rest of Canada) | | |  | |
| 2005 | | Statistics Canada  Cross-sectional | Social determinants | | | 2 | CCHS 3.1 (2005) | | | Canada  n = 67,741  age: 12+  m/f: not given  general pop’n | |  | | Sense of community belonging, region, socio-demographic factors | | | Feelings of community belonging were associated with substantially better SRMH | | |  | |
| 2006 | | Shields, M.  Cross-sectional | Social determinants | | | 2 | CCHS 1.2 (2002) | | | Canada  n = 20,747  age: 18-75  m/f: not given  general pop’n | | stress | | Job satisfaction, SRH, gender, age | | | F/p SRMH & SRH associated with low job satisfaction. Association for SRMH stronger. | | |  | |
| 2007 | | Mulvaney-Day, N.E.*, et al.*  Cross-sectional | Social determinants | | | 2 | NLAAS (2002) | | | USA (Latino)  n = 2,554  age: 18+  m/f: 1/1  Latino American pop’n | |  | | SRH, family support, family cultural conflict, ethnicity; nativity; English prof.; socio-demographic factors | | | Family support & family cultural conflict were strongly associated with SRMH & SRH | | |  | |
| *Objective for using SRMH: “1” as a minor variable; “2” as a major variable; “3” as the subject of a validation study | | | | | | | | | | | | | | | | | | | |  | |
| 2009 | | Zhang, W.  Cross-sectional | Social determinants | | | 2 | NLAAS (2002) | | | USA  n = 2034  age: 18-65+  m/f: 1/1  Asian Amer pop’n | |  | | SRH, socioeconomic status, immigration factors, social connections, family cohesion | | | Differences in SRMH exist depending on country of origin; patterns of association vary; social connection measures are mediated by SES and immigration factors. | | |  | |
| 2009 | | Perera, B., *et al.*  Cross-sectional | Social determinants | | | 2 | Primary data collection | | | Sri Lanka  n = 2,077  age: 18-85  mean age: 40.16  m/f: 1/1.2  gen pop in Southern Sri Lanka | | Stressors (nuisance from neighbours, nuisance from drug users, shortage of water, poor water/sewage drainage) | | SRH | | | The stressors listed were all associated with SRMH. None were associated with SRH. | | |  | |
| 2010 | | De Castro, *et al.*  Cross-sectional | Social determinants | | | 2 | NLAAS (2002) | | | USA  n =1,181  age mean: 41  m/f: 1/1  Latino/Asian Amer pop’n | |  | | SRPH; employment frustration, English proficiency | | | Negative association between employment frustration and SRMH. | | |  | |
| 2010 | | Maximova, K. & Krahn, H.  Cohort | Social determinants | | | 2 |  | | | Canada  n =525  age: 15+  mean age: 36.7  m/f: 1/1  pop’n: refugees in Alberta | | Changes in SRMH | | SRPH; changes in SRPH; employment history, education, refugee experiences, settlement services | | | Current employment and access to settlement services associated with improvements in SRMH. Time in a refugee camp and having held a professional job in home country associated with decline in SRMH. | | |  | |
| 2011 | | Veenstra, G.  Cross-sectional | Social determinants  Physical health | | | 2  1 | Primary data collection | | | Canada  n = 1499  age: 19+  m/f: 1/2  general pop’n | | Depressive feelings | | Conceived racial identity, medical conditions | | | Mismatched racial identities correspond with relatively high risks of various poor health outcomes. | | |  | |
| *Objective for using SRMH: “1” as a minor variable; “2” as a major variable; “3” as the subject of a validation study | | | | | | | | | | | | | | | | | | | |  | |
| 2012 | | Lam, J., *et al.*  Cross-sectional | Social determinants | | | 2 | NLAAS (2002) | | | USA  n =1639  age: 18-96, mean mean age: 42.3  m/f: 1/1  Asian American pop’n | | Acculturation stress | | SRPH, age, age of immigration, perceived differences in social status, gender, region, income. | | | No effects were observed for mental health. | | |  | |
| 2012 | | Schacter, A., *et al.*  Cross-sectional | Social determinants | | | 2 | NLAAS (2002) | | | USA  n =3,264  age: 18-97  mean age:43.4  m/f: 1/1  pop’n: Latino-Asian American | |  | | SRPH, language proficiency, socioeconomic status, family support | | | Bilingualism associated with better SRH and SRMH; associations are partially mediated by socioeconomic status and family support. | | |  | |
| 2012 | | John, D., *et al.*  Cross-sectional | Social determinants | | | 2 | NLAAS (2002) | | | USA  n =1530  age: 18+  mean age: 39  m/f: 1/1  dx: MH  pop’n: Latino-Asian American | | Mental disorder based on DSM-IV ; acculturation stress | | SRPH, socioeconomic factors, English prof, financial need, social support, discrimination, nativity. | | | Compared to U.S.-born Asians, immigrants had increased odds for reporting fair/poor mental health and decreased odds for any DSM-IV mental disorder and anxiety; age and gender were controlled. The occupational class-health gradient was not strong. | | |  | |
|  | *Objective for using SRMH: “1” as a minor variable; “2” as a major variable; “3” as the subject of a validation study | | | | | | | | | | | | | | | | | | | | |
|  | **Abbreviations:**  BASIS-24: Behavior and Symptom Identification Scale 24  CCHS: Canadian Community Health Survey  CES-D: Center for Epidemiologic Studies Depression scale  DSM: Diagnostic and Statistical Manual for mental disorders  dx: diagnosis  f/p: fair or poor;  gen pop’n: general population | | | | | | | | GP: general practitioner  GDS-SF: Geriatric Depression Scale - Short Form  K6: Kessler Psychological Distress Scale  m/f: male or female  MCS: Mental Component Survey  MEPS: Medical Panel Expenditure Panel Survey  MH: mental health;  NLAAS: National Latino and Asian American Study | | | | | | PHQ: Patient Health Questionnaire  QOL: Quality of Life  SF-12: Short Form -12 items  SRMH: Self-rated mental health  SRH: Self rated health  SRPH: Self rated physical health  WHM-CIDI: World Health Mental Composite  International Diagnostic Interview | | | | | |  |
